# Mobile digital health devices and the diagnosis in real-time of myocardial ischaemia

**DOI:** 10.1093/qjmed/hcx046

**Published:** 2017-02-28

**Authors:** J.P. Joseph, S.R. Redwood

**Affiliations:** From the King’s College London British Heart Foundation Centre of Excellence, The Rayne Institute, St. Thomas’ Hospital Campus, London, UK

Learning points for clinicians

- Mobile digital health devices that provide remote, patient-generated information are commercially available. This can challenge traditional health care delivery models, but can potentially reduce healthcare expenditure and improve clinical outcomes. Previous, difficult to establish diagnoses, such as coronary artery spasm, may be made with greater certainty using these devices.

A 61-year-old man presented with sub-sternal chest pain at rest associated with sweating. He had a family history for premature cardiovascular disease, but otherwise no cardiac risk factors and an excellent exercise tolerance. The symptoms had been recurrent for 3 months, and on one occasion he presented to the emergency department where both 12-lead electrocardiogram (ECG) and troponin were normal. He was prescribed GTN spray, and following cardiology review went to have a normal exercise stress echocardiogram.

He continued to experience episodes of chest discomfort which resolved with GTN administration, during one of these episodes he acquired a tracing using a handheld ECG device (AliveCor Inc., USA) attached to a smartphone. The single ECG lead tracing demonstrated ST-segment elevation with intermittent ectopy (Figure 1A) which resolved following the administration of GTN (Figure 1B).

**Figure 1:**
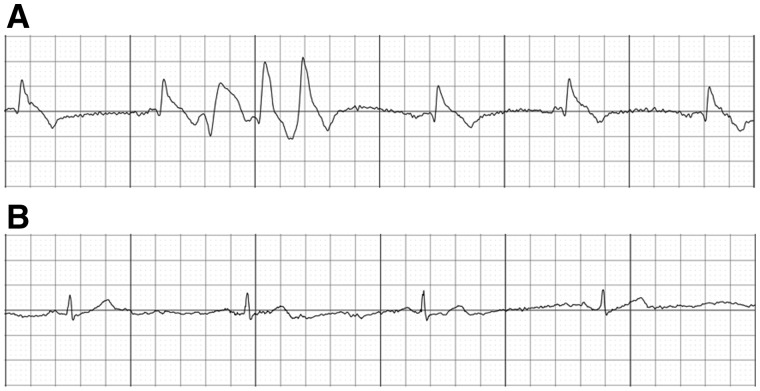
Single-lead ECG recordings obtained with the AliveCor device during a period of chest pain at rest demonstrated ST-elevation and an ectopic triplet (**A**) and following sublingual GTN administration normalization of the ST segments (**B**)—both traces obtained with mains filter: 50 Hz; Scale: 25 mm/s, 10 mm/mV.

Due to the transient ST-segment elevation, he was admitted to hospital and underwent urgent coronary angiography. This demonstrated minor atheromatous changes which, following pressure wire assessment using intravenous adenosine, were demonstrated not to be physiologically significant. Given the absence of exertional symptoms, normal stress echocardiogram and invasive assessment, the decision was made not to proceed to angioplasty.

A diagnosis of coronary artery spasm was made and treatment with vasodilators (diltiazem) and statin initiated. At 6 months clinic follow-up, the patient was asymptomatic with excellent exercise capacity.

## Discussion

Recent technological advances have resulted in a wide range of mobile digital health (mHealth) devices that are disrupting traditional health care delivery. These miniaturized diagnostic instruments are providing remote, patient-generated information that can potentially reduce healthcare expenditure and improve clinical outcomes.^1^

AliveCor incorporates electrodes for wireless cardiac telemetry monitoring, and approved for use by the US Food and Drug Administration (US-FDA) and EU Medical Device Directive (EU-MDD) in 2013. When the user rests their fingers on the sensors, the device detects electrical impulses (representative of Lead I when held between the right and left hand) and converts it to an ultrasound signal that is transmitted to the mobile device’s microphone to produce a 30 s rhythm strip. Previous studies have established clinical effectiveness in detecting arrhythmias, but this is the first reported case of mHealth products identifying acute myocardial ischaemia.^2^

In 1959, Prinzmetal *et al.* described ‘variant angina’ and postulated that the condition was due to vasospasm.^3^ The pathophysiology of coronary artery spasm remains unclear, but is thought to involve endothelial dysfunction and hyper-reactivity of coronary artery smooth muscle cells. Classically, this presents with severe chest pain, usually at rest, with a concurrent ECG showing transient ST elevation and prompt resolution with short-acting nitrates—all of which are demonstrated the our reported case.^4^

Contemporary diagnosis of coronary artery spasm relies on provocation testing with intracoronary acetylcholine during invasive coronary angiography—although this remains a controversial area. Many cardiologists have questioned the clinical utility of provocation testing as the results are difficult to interpret and studies have demonstrated high proportions of patients with provoked spasm during invasive testing.^5^ The gold standard for diagnosis would be demonstrating transient ST-elevation during episodes of vasospasm in a patient with unobstructed coronary arteries. This has proven elusive due to the unpredictable nature of symptoms and the impracticality of prolonged rhythm monitoring.

This reported case provides a succinct demonstration how emerging mHealth devices can provide new insights into previously difficult to establish diagnoses. These devices are providing patients with accurate diagnostic tools that have the potential to challenge the way we practice medicine. Future work will focus on how best to analyse these vast quantities of data, identifying clinically useful measures and ways to integrate them into our existing healthcare systems to improve patient outcomes.
